# Impact of MERS-CoV and SARS-CoV-2 Viral Infection on Immunoglobulin-IgG Cross-Reactivity

**DOI:** 10.3390/vaccines11030552

**Published:** 2023-02-26

**Authors:** Joud Mohammed AlKhalifah, Waleed Seddiq, Mohammed Abdullah Alshehri, Abdulkarim Alhetheel, Ahmed Albarrag, Sultan Ayoub Meo, Jaffar A. Al-Tawfiq, Mazin Barry

**Affiliations:** 1 College of Medicine, King Saud University, Riyadh 11461, Saudi Arabia; 2 Center for Stem Cell and Translational Immunotherapy (CSTI), Harvard Medical School, Boston, MA 02115, USA; 3 Department of Neurosurgery, Brigham and Women’s Hospital, Boston, MA 02115, USA; 4 Department of Pathology, College of Medicine, King Saud University, Riyadh 11461, Saudi Arabia; 5 Department of Physiology, College of Medicine, King Saud University, Riyadh 11461, Saudi Arabia; 6 Department of Medicine, Johns Hopkins University School of Medicine, Baltimore, MD 21205, USA; 7 Infectious Disease Division, Department of Medicine, School of Medicine, Indiana University, Indianapolis, IN 46202, USA; 8 Department of Medicine, Division of Infectious Diseases, College of Medicine, King Saud University, Riyadh 11461, Saudi Arabia

**Keywords:** SARS-CoV-2, MERS-CoV, cross-immunity, immunity, coronaviruses

## Abstract

Severe Acute Respiratory Syndrome Coronavirus 2 (SARS-CoV-2) has posed a considerable threat to public health and global economies. SARS-CoV-2 has largely affected a vast world population and was declared a COVID-19 pandemic outbreak, with a substantial surge of SARS-CoV-2 infection affecting all aspects of the virus’ natural course of infection and immunity. The cross-reactivity between the different coronaviruses is still a knowledge gap in the understanding of the SARS-CoV-2 virus. This study aimed to investigate the impact of MERS-CoV and SARS-CoV-2 viral infections on immunoglobulin-IgG cross-reactivity. Our retrospective cohort study hypothesized the possible reactivation of immunity in individuals with a history of infection to Middle East Respiratory Syndrome coronavirus (MERS-CoV) when infected with SARS-CoV-2. The total number of participants included was 34; among them, 22 (64.7%) were males, and 12 (35.29%) were females. The mean age of the participants was 40.3 ± 12.9 years. This study compared immunoglobulin (IgG) levels against SARS-CoV-2 and MERS-CoV across various groups with various histories of infection. The results showed that a reactive borderline IgG against both MERS-CoV and SARS-CoV-2 in participants with past infection to both viruses was 40% compared with 37.5% among those with past infection with MERS-CoV alone. Our study results establish that individuals infected with both SARS-CoV-2 and MERS-CoV showed higher MERS-CoV IgG levels compared with those of individuals infected previously with MERS-CoV alone and compared with those of individuals in the control. The results further highlight cross-adaptive immunity between MERS-CoV and SARS-CoV. Our study concludes that individuals with previous infections with both MERS-CoV and SARS-CoV-2 showed significantly higher MERS-CoV IgG levels compared with those of individuals infected only with MERS-CoV and compared with those of individuals in the control, suggesting cross-adaptive immunity between MERS-CoV and SARS-CoV.

## 1. Introduction

Severe Acute Respiratory Syndrome Coronavirus 2 (SARS-CoV-2) has caused a great threat to public health and global economies [1]. The human “coronaviruses” (HCoV) are large, enveloped RNA viruses belonging to the family Coronaviridae [1,2] that cause various respiratory illnesses [3], including the common cold [4], influenza-like illness (ILI) [5], and acute respiratory illnesses (ARI) [6], such as pneumonia [7], exacerbations of underlying lung disease [8], and bronchiolitis [9]. In December 2019, the novel beta coronavirus, SARS-CoV-2, was initially found in Wuhan, China, and has become a pandemic virus. By 27 January 2023, SARS-CoV-2 had resulted in at least 752,517,552 confirmed cases and 6,804,491 deaths, with a mortality rate of 0.90% [10], and had caused significant socioeconomic implications globally [11,12].

Before the present COVID-19 pandemic, the world faced another similar infectious respiratory disease in the Middle East region known as “Middle East Respiratory Syndrome Coronavirus (MERS-CoV)”. After the disease was first identified in April 2012, it was reported for the first time in a patient who died due to a lower respiratory tract infection in Jeddah, Kingdom of Saudi Arabia (KSA) [13,14]. Since that time, 2,600 cases of MERS-CoV have been reported from 27 states worldwide. From January 2022 through 22 January 2023, six MERS-CoV cases have also been reported in the Middle East region as follows: three in Saudi Arabia, two in Qatar, and a single case in Oman, among which the outcome was one death, with a fatality rate of 16.6% [15]. MERS-CoV, which is still endemic in the region, has caused recurrent community outbreaks, and its involvement with severe disease and high mortality rates continues to cause global public health concerns [16].

During the COVID-19 pandemic, the emergence of SARS-CoV-2 variants with different transmission trends has been observed. However, the various variants’ effect on the population and the immunity against novel SARS-CoV-2 may be influenced by prior exposures to related viruses, such as SARS-CoV variants, MERS-CoV, and seasonal coronaviruses, along with the level of cross-reactive immunity conferred by those exposures. Novel variants may be more infectious than the previous virus to invade the population. However, in a more realistic scenario in which cross-reactive immunity is partial, the novel variants can invade, even if they are less transmissible than previously circulating viruses. This is because partial cross-reactive immunity effectively increases the pool of susceptible hosts that are available to the novel variant compared with that of complete cross-reactive immunity [17]. Once the earlier infection with the antigenically related virus assists the establishment of infection with the novel variant, then even variants with very limited transmissibility can invade the host population [17].

The substantial surge of SARS-CoV-2 infection during recent years, grasping all aspects of knowledge about this virus’ natural course of infection and immunity was eminent. Cross-immunity between coronaviruses still presents a gap in knowledge in understanding the virus. Limited literature highlights the results with a substantial amount of cross-reactivity and recognition by the host’s immune response between different coronavirus infections [18]. “SARS-CoV-1, MERS-CoV, and SARS-CoV-2 share significant sequence homology and potentially share antigenic epitopes capable of inducing an adaptive immune response”. Excitingly, it was noticed that there is a high-level degree of “cross-reactivity between T- and B-cell epitopes, and antibodies produced against the relevant viral structural proteins of SARS-CoV-2 and other SARS-like viruses” [19]. The evidence on cross-serologic reactivity between MERS-CoV and SARS-CoV-2 is deficient. Thus, our study aimed to investigate and compare the humoral immune response against MERS-CoV and SARS-CoV-2.

## 2. Subjects and Methods

### 2.1. Study Design and Settings

This cross-sectional study was conducted at the College of Medicine, King Saud University, Riyadh, Saudi Arabia. The total number of participants included in this study was 34; among them, 22 (64.7%) were males, and 12 (35.29%) were females. The mean age of the participants was 40.3 ± 12.9 years. After receiving signed consent, a 5cc sample of blood in a yellow-topped tube was collected by the venipuncture method. The serum samples were accumulated, anonymized, aliquoted, and stored at −80 °C until analyzed. The entire research methodology and procedures were conducted as per the ethical standards of the King Saud University ethical committee, IRB-E-21-5849, for de-identified samples for method development and in accordance with the Helsinki Declaration.

### 2.2. Study Participants, Inclusion, and Exclusion Criteria

Participants 18 years and older were included in this study by the convenience sampling technique. Initially, they were recruited based on four groups: controls with no previous infection with SARS-CoV-2 or MERS-CoV (C-Gp), laboratory-confirmed infection with SARS-CoV-2 only (SV-Gp), laboratory-confirmed infection with MERS-CoV only (MV-Gp), and laboratory-confirmed infection with both SARS-CoV-2 and MERS-CoV (SV-MV-Gp).

All participants were recruited based on the presence of a laboratory-confirmed, real-time reverse transcriptase polymerase chain reaction (PCR) infection test done by a nasopharyngeal swab. Participants with a past infection of MERS-CoV between January 2014 to December 2018 were recruited, while participants with a past infection of SARS-CoV-2 between March 2020 to September 2021 were recruited.

Any participant with only a clinical history of infection without a PCR confirmation was excluded, as well as individuals younger than 18 years old. Participants with a known immunological disease and/or on immunosuppressive therapy and/or recently diagnosed with malignancy were excluded.

### 2.3. Detection of MERS-CoV-IgG Antibodies Using Enzyme-Linked Immunosorbent Assay

The blood samples were assayed using the MERS-CoV- IgG enzyme-linked immunosorbent assay (ELISA) kit (Euroimmun, Luebeck, Germany), following the manufacturer’s instructions. The test is designed to “detect IgG antibodies specific to the S1 antigen of MERS-CoV in human serum. The capacity of recognition of this kit, as specified by the company, is a ratio of 0.04. A sample is considered negative if the ratio <0.80, positive if the ratio >1.10, or equivocal if the ratio >0.80 and <1.10”.

### 2.4. Detection of SARS-CoV-2-IgG Antibodies Using Enzyme-Linked Immunosorbent Assay

The blood samples were examined using the SARS-CoV-2 IgG enzyme-linked immunosorbent Quantitative assay (ELISA) kit II (Abbott, Chicago, IL, USA) in accordance with the manufacturer’s instructions. The “SARS-CoV-2 IgG II Quant assay is an automated, two-step chemiluminescent microparticle immunoassay (CMIA). It is used for the qualitative and quantitative determination of IgG antibodies to the receptor binding domain (RBD) of the S1 subunit of the spike protein of SARS-CoV-2 in human serum and plasma on the Alinity system. The sequence used for the RBD was taken from the WH-Human 1 coronavirus, GenBank accession number MN908947”. The maximum level of the detection of the kit, as suggested by the manufacturer, is the interval stated as 21 to 40,000 AU/mL, and the positivity cutoff is ≥50 AU/mL [20].

### 2.5. Statistical Analysis

The data were entered and analyzed by using the SPSS 26.00 statistical software. The descriptive statistics (mean, standard deviation, and median), along with the chi-square, were used to describe quantitative data, and frequencies and percentages were used to describe qualitative data. A *p*-value less than 0.05 was considered significant.

## 3. Results

### 3.1. Demographics and Distribution

The total number of participants included in this study was 34; of those, 22 (64.7%) were men, and 12 (35.29%) were women. The mean age of the participants was 40.3 ± 12.9 years. Of the included participants, 13 (38.23%) were in C-Gp (control), 8 (23.52%) were in SV-Gp, 8 (23.52%) were in MV-Gp, and 5 (14.7%) were in SV-MV-Gp. Most of the participants’ educational levels were college degrees or above (a total of 22 participants (64.70%)). Regarding occupation, 13 (41.17%) were healthcare workers, while 21 (61.76%) were non-healthcare workers. Thirty-three participants (97%) were vaccinated with at least two doses of the SARS-CoV-2 vaccine, as seen in Table 1.

All other demographic factors were not explained, since our research showed no statistical significance for age, gender, comorbidities, and occupation with the levels of MERS-CoV IgG, corresponding with the *p*-values 0.395, 0.532, 0.409, and 0.261, respectively. In contrast, the only demographic factor with influence on the outcome was the level of education, with a *p*-value of 0.0001.

### 3.2. Humoral Immune Response to SARS-CoV-2

**Figure 1:**
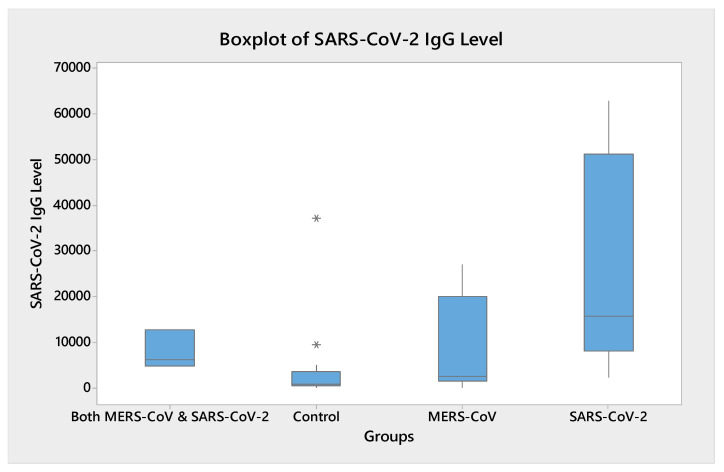
SARS-CoV-2 Serology as Boxplot.

There were eight participants (23.52%) who were recruited in the study with a history of infection to SARS-CoV2 (SV-Gp), and five participants (14.7%) with a history of infection of both SARS-CoV-2 and MERS-CoV (SV-MV-Gp). Among these, 13 (100%) were vaccinated with two doses of the COVID-19 vaccine, and 3 (23%) were vaccinated with three doses, as illustrated in Figure 1. 

### 3.3. Humoral Immune Response MERS-CoV 

**Figure 2:**
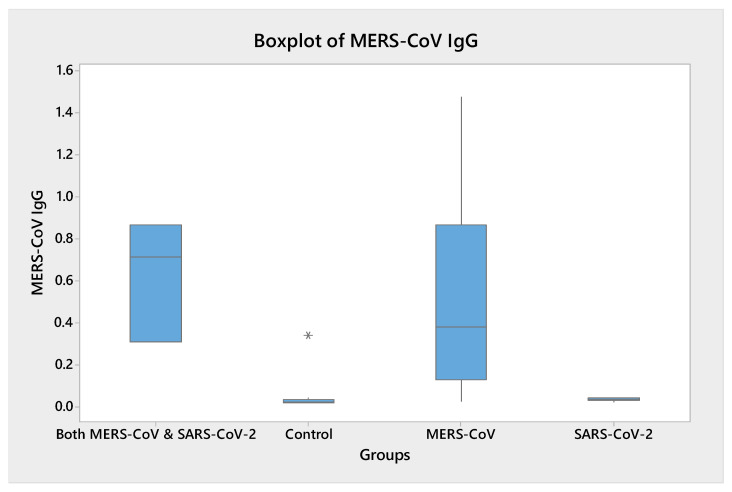
MERS-CoV Serology as Boxplot.

As shown in Figure 2, the IgG levels against MERS-CoV varied by groups, as seen in Table 2. None of the participants in the SV-Gp and control had reactive IgG, while there were three (37.5%) positive/borderline cases among MV-Gp compared with two (40%) among SV-MV-Gp.

### 3.4. Cross-Immunity between SARS-CoV-2 and MERS-CoV

In SV-MV-Gp, the percentage of those who had positive borderline IgG for MERS-CoV was 40% compared with 37.5% in the MV-Gp. The correlation between the levels of MERS-CoV IgG and infection with both SARS-CoV-2 and MERS-CoV showed a statistical link, with a *p*-value equal to 0.0001.

## 4. Discussion

Since December 2019, the SARS-CoV-2 outbreak has developed a threatening impact on the global healthcare system and the economies. The disease is highly contagious and swiftly spread across the world [1]. There is a great discussion about the human immune response to novel coronaviruses and cross-reactive immunity between SARS-CoV-2 and earlier exposure to other viruses such as MERS-CoV. The immune system remains the definitive defense system supporting the human body to fight against various pathogens. Innate immunity is the first line of defense against various infections, including the SARS-CoV-2 and MERS-CoV diseases [21].

The SARS-CoV-2 and MERS-CoV diseases are highly contagious and have affected a huge population worldwide. The coronavirus has diverse epidemiological and biological characteristics. Our study demonstrates that individuals with previous infections with both MERS-CoV and SARS-CoV-2 showed significantly higher MERS-CoV IgG levels compared with levels in those infected only with MERS-CoV and compared with levels in the control, suggesting cross-adaptive “immunity between the MERS-CoV and SARS-CoV”. There are seven coronaviruses linked with pathologies in humans, which mostly cause a mild degree of respiratory ailment. However, “SARS-CoV-1, MERS-CoV, and SARS-CoV-2” cause substantial mortality [22]. The variation in the epidemiology of SARS-CoV-2 cases and deaths in the various regions around the world may be due to variable adaptive immune responses due to prior exposure to coronaviruses [23]. The three viruses “SARS-CoV-1, MERS-CoV and SARS-CoV-2 share significant sequence homology, and potentially share antigenic epitopes capable of inducing an adaptive immune response”. This may be a possible cause that previous exposure to one virus could confer partial immunity to another virus [24].

The literature acknowledges that an individual with a previous MERS-CoV infection can exhibit a cross-reactive immune response to a SARS-CoV-2 infection [25]. Kim et al., 2020 [26] and Al Maani et al., 2021 [19] found antibodies against spike proteins with previous MERS-CoV three years after the infection. In another, the authors detected the MERS-CoV–specific neutralizing antibodies six years post-infection in patients with earlier exposure to MERS-CoV infection. It has also been reported that previous MERS-CoV infection can reveal cross reactive response to SARS-CoV-2 disease [19,26].

The Asian and Middle Eastern populations, who experienced repeated exposure to multiple rounds of coronavirus infections, may build up an adaptive immune response to SARS-CoV-2, limiting infection and preventing reinfection by producing neutralizing antibodies through the humoral immune system. IgG production is mainly caused by SARS-CoV-2 infection, which produces an N protein that can be detected as soon as day four of acquiring the infection, with most individuals seroconverting by day 14 [27]. The “IgG specific and neutralizing antibodies were detected two years after the SARS-CoV-1 infection by immunofluorescence assays and ELISA in 90% of recovered patients” [27]. Moreover, “peak-specific IgM on the ninth day after the disease and the class switching to IgG in the second week were detected” [28]. During a long-term follow-up of survivors, IgG was exclusively measurable in patients who recovered almost six years after the SARS-CoV-2 infection, indicating diminishing levels of memory B-cells would also be found against SARS-CoV-2 [28,29]. In spite of these protective roles for “T-cells in MERS-CoV infection, others have found that depletion of CD8 T cells in a sublethal mouse model of MERS-CoV results in diminished lung pathology and clinical disease without impacting the viral titers, suggesting that these cells may play a role in immunopathogenesis” [29].

Evidence of pre-existing humoral cross-reactive immunity to SARS-CoV-2 was also raised by Mveang Nzogh et al. [30], who examined the samples of healthy volunteers in 2014. Of 135 samples, 32 (23.7%) were confirmed positive for antibodies against SARS-CoV-2 N. This immunity can be attributed to several factors, among which mostly cross-immunity between coronaviruses seems accountable. MERS-CoV-specific antibodies were reported to persist for at least two years in patients who recovered from the infection, and “memory T cell responses among survivors were polyfunctional, expressing both IFN-γ and TNF, consistent with the greater protective ability”. These responses may be detectable in patients as late as two years post-infection, including in patients with no detectable antibody response. It suggests that immune memory remains intact despite transient antibody responses. 

Zhao et al. [31] reported that intranasal vaccination with Venezuelan equine encephalitis replicons “(VRP) encoding a SARS-CoV-1 N protein CD4 T cell epitope resulted in some degree of cross-protection against MERS-CoV, which showed reduced viral load. This epitope is well conserved between these two coronaviruses and related bat coronaviruses. It was also observed that mice immunized with the MERS-CoV-specific epitope mediated some cross-protection against SARS-CoV-1 infection, and the homologous epitope in a MERS-like bat coronavirus (HKU4) mediated protection against MERS-CoV challenge”.

The literature shows that the immune reactivity to SARS-CoV-2 in individuals that were infected before the COVID-19 pandemic provides conclusive evidence that SARS-CoV-2 cross-reactive immune responses may be derived from non-SARS-CoV-2 antigens. Mbow et al., 2020 [32] conducted a study and reported a mystery about the trends of the COVID-19 pandemic. The authors identified relatively low COVID-19 cases and deaths in the sub-Saharan region [33]. However, the USA and many European countries have experienced more confirmed cases, and epidemiological trends were extended to several countries worldwide. There are multiple aspects, including climate, weather conditions, and a young adult population, that can contribute to the decreased occurrence of COVID-19 cases and deaths in the regions of Sub-Saharan Africa, although the age-allied protective factor may decrease with various COVID-19 variants, which circulate widely. The proof exists whether the responses protect against COVID-19 or not. The literature also supports the hypothesis that factors that may be related to the decreased number of COVID-19 cases and deaths in Sub-Saharan Africa may be due to the earlier exposure to coronaviruses that may induce cross-protective immunity.

Similarly, Borrega et al., 2021 [33] examined the blood samples obtained from the population of Sierra Leoneans that were gathered before the appearance of the first case of COVID-19 in late 2019. It was reported that during the COVID-19 pandemic, Sierra Leoneans most likely had more frequent exposures to coronaviruses with SARS-CoV-2, SARS-CoV, and MERS-CoV than Americans did. The possible justification for these findings is that the immunological repertoires produce more widely cross-reactive antibodies on coronavirus infection. The authors further reported that immunity to coronavirus may be a possible factor in minimizing the burden of cases and deaths from COVID-19. It is expected that people are frequently exposed to SARS-CoV and MERS-related viruses. It is also possible that the cellular immunity to endemic coronaviruses also has a protective role against COVID-19.

The earlier literature also highlighted the possibility for the diseases with seasonal coronaviruses to produce cross-reactive immune responses to SARS-CoV-2. The antibodies to “SARS-CoV and MERS-CoV can cross-react with various SARS-CoV-2 antigens”. Anderson et al. [34] and Ng et al. [35] found the SARS-CoV-2-neutralizing antibodies in pre-pandemic blood samples. The authors reported that the sera from SARS-CoV-2 of uninfected children or adolescents neutralized SARS-CoV-2. The contradictory findings were recorded on whether the pre-existing humoral immunity induced by infection with seasonal coronaviruses confers protection against the SARS-CoV-2. Some studies have found that pre-existing immunity against endemic human coronaviruses was not associated with protection against SARS-CoV-2 infections [34]. 

Hypothetically, the prior immunity to a SARS-CoV in the Sierra Leonean population may mitigate against the spread of SARS-CoV-2. A similar hypothesis was proposed by Tso et al. [36] in the analysis of pre-pandemic blood samples from Tanzania and Kenya. The pre-existing immunity is currently not well understood in counteracting the efforts at vaccine delivery, which can be less protective against the Delta variant than other SARS-CoV-2 variants. The cross-reactive immune responses that occur in people may impact the susceptibility to infection and disease severity. With the high levels of similarity of SARS-CoV-2 with other HCoVs, it may be possible that the viruses could be the main cause of cross-reactive immune responses. However, the particular mechanism is still not well-established, and the precise source and the resultant role of pre-existing SARS-CoV-2 cross-reactivity remain a challenging question of interest for the research community. Over the last two decades, particularly since mid-2002, the three extremely infective and deadly human coronaviruses “(β-hCoVs), namely, SARS-CoV, MERS-CoV, and SARS-CoV-2, have globally emerged and culminated in the occurrence of SARS epidemic, MERS outbreak, and coronavirus disease 19 (COVID-19) pandemic, respectively”. These viruses have developed into pandemic situations worldwide. The cross-reactivity between the different coronaviruses is still a knowledge gap in the understanding of the SARS-CoV-2 virus. This study provides a better understanding of the impact of MERS-CoV and SARS-CoV-2 viral infections on immunoglobulin-IgG cross-reactivity [37,38].

The literature demonstrates the resemblances in the immune responses among the SARS-CoV-2 and MERS diseases, specifically the T cell response [37,38]. The global research community hypothesized the possible role of cross-reactive immunity between the pathogens. People with prior exposure to the MERS-CoV disease can demonstrate a cross-reactive immune response to a SARS-CoV-2 infection. The prevalence of SARS-CoV-2 was decreased in people with a previous MERS-CoV infection more than it was decreased in MERS-CoV-negative individuals [39].

In our perspective of constant infections with various SARS-CoV-2 variants, it is expected that cross-reactive memory responses to SARS-CoV-2 variants and COVID-19 vaccines can create a cross-reactive response that may provide limited protection from future various variants. There is the possibility that the arrangement of various infectious diseases may play a part in assessing the effectiveness of the immune response to counteracting the SARS-CoV-2 disease. The vaccines campaign against SARS-CoV-2 develop immunity and offer a way to protect people from future coronavirus pandemics.

### Study Strengths and Limitations

This is the first study to examine cross-immunity between MERS-CoV and SARS-CoV-2 in a MERS-CoV-endemic country, among 2014–2018 MERS survivors, detecting their serological status compared to different control groups. This study has some limitations due to the convenience sampling procedure; representativeness was difficult due to the overall small size and the procedure being conducted in a single center. In this study, the total number of participants was 34; this sample size was further decreased when the participants were divided into groups with a history of SARS-CoV2 (SV-Gp) infection and with a history of both SARS-CoV-2 and MERS-CoV (SV-MV-Gp) infections. In future, such studies will be conducted at multicenter and large-scale levels, along with measuring antibodies and levels of B memory cells to reach better conclusions.

## 5. Conclusions

Our study demonstrates that individuals with previous infections with both MERS-CoV and SARS-CoV-2 showed significantly higher MERS-CoV IgG levels compared with those of individuals infected only with MERS-CoV and compared with those of the control, suggesting cross-adaptive immunity between MERS-CoV and SARS-CoV. The findings support the evidence that cross-reactive immunity from common human coronaviruses can influence the immunological response to SARS-CoV-2. It is suggested that further large-sample-sized studies should be conducted to fully understand and characterize the immune responses directed against coronaviruses.

## Figures and Tables

**Table 1:** The demographic characteristics of the participants (n = 34).

Demographic Characteristics	Number (Percentage %)
Sex	
Male	22 (64.70%)
Female	12 (35.29%)
Age	
18–24	4 (11.76%)
25–34	11 (32.35%)
35–44	10 (29.41%)
45–54	4 (11.76%)
55–64	3 (8.82%)
65–74	2 (5.88%)
Educational Level	
College degree or higher level	22 (64.70%)
High school	8 (23.52%)
Middle school	2 (5.88%)
Primary school	2 (5.88%)
Residence Area	
Riyadh	29 (85.29%)
Al Qassim	5 (14.70%)
Occupation	
Healthcare worker	13 (41.17%)
Non-healthcare worker	21 (61.76%)
Comorbidities (diabetes mellitus, hypertension, and/or dyslipidemia)	
Yes	12 (35.29%)
No	22 (64.70%)
Vaccine Doses	
Vaccine Dose-1	
Pfizer	26 (76.47%)
AstraZeneca	8 (23.52%)
Vaccine dose 2	
Pfizer	26 (76.47%)
AstraZeneca	7 (20.58%)
Vaccine Dose 3	
Pfizer	7 (20.58%)
AstraZeneca	1 (2.94%)
Infection history	
Control with no previous infection with SARS-CoV-2 or MERS-CoV (C-Gp)	13 (38.23%)
Infection with MERS-CoV only (MV-Gp)	8 (23.52%)
Infection with SARS-CoV-2 only (SV-Gp)	8 (23.52%)
Infection with both SARS-CoV-2 and MERS-CoV (SV-MV-Gp)	5 (14.70%)

**Table 2:** SARS-CoV-2 IgG and MERS-CoV IgG levels across the various groups.

Groups	Number of Days (ds) Since Last Vaccination and Collection of Samples	Number of SARS-CoV-2 Vaccines	SARS-CoV2 IgG Levels (AU/mL)	Mean (AU/mL)	Date of MERS-CoV/SARS-CoV2 Infection Where Applicable		MERS-CoV IgG Levels. (Ratio)
Control (No previous infection with SARS-CoV-2 or MERS-CoV (C-Gp))				4679			
No. 1	(Third dose) 184 ds	3	930.1		N/A		0.0224
No. 2	(Second dose) 658 ds	2	9460.6		N/A		0.0176
No. 3	(Second dose) 143 ds	2	1107.2		N/A		0.04
No. 4	(Second dose) 84 ds	2	364.8		N/A		0.3424
No. 5	(Second dose) 157 ds	2	860.9		N/A		0.0352
No. 6	(Second dose) 156 ds	2	5078.1		N/A		0.0224
No. 7	(Second dose) 93 ds	2	635.6		N/A		0.024
No. 8	(Second dose) 141 ds	2	2064.6		N/A		0.0176
No. 9	(Second dose) 63 ds	2	37,272.1		N/A		0.0224
No. 10	(Second dose) 157 ds	2	269.1		N/A		0.0256
No. 11	(Second dose) 157 ds	2	1626.4		N/A		0.0432
No. 12	(Second dose) 188 ds	2	639.1		N/A		0.0192
No. 13	(Second dose) 288 ds	2	518.3		N/A		0.0176
Infection with SARS-CoV-2 only (SV-Gdsp)				27,854			
No. 1	(Third dose) 150 ds	3	10,621.3		6/10/2020		0.0368
No. 2	(Second dose) 100 ds	2	7369.8		9/2/2020		0.032
No. 3	(Second dose) 112 ds	2	19,165.2		6/30/2021		0.0464
No. 4	(Second dose) 123 ds	2	2380.2		5/14/2020		0.032
No. 5	(Third dose) 41 ds	3	62,790.1		9/23/2021		0.0448
No. 6	(Second dose) 65 ds	2	12,164.3		7/15/2020		0.0336
No. 7	(Third dose) 53 ds	3	56,728.8		7/14/2020		0.0448
No. 8	(First dose) 132 ds	1	34,380.4		9/2/2020		0.024
Infection with MERS-CoV only (MV-Gp)				10,292			
No. 1	(Second dose) 90 ds	2	2807.1		1/17/2016		0.0336
No. 2	(Second dose) 256 ds	2	1503.1		11/20/2018		0.9504 (Borderline)
No. 3	(Second dose) 23 ds	2	26,955.9		9/20/2014		0.0288
No. 4	(Third dose) 55 ds	3	19,770.4		4/29/2014		0.2368
No. 5	(Second dose) 159 ds	2	1589		8/15/2015		0.3264
No. 6	(Second dose) 523 ds	3	2335.7		12/2/2015		0.5872
No. 7	(Third dose) 52 ds	2	21,158.1		9/25/2015		0.84 (Borderline)
No. 8	(Second dose) 99 ds	2	6219.3		3/4/2016		1.4768 (Pos)
Both SARS-CoV-2 and MERS-CoV (SV-MV-Gp)				7986	SARS-CoV2	MERS-CoV	
No. 1	(Second dose) 44 ds	2	6272.4		7/13/2021	6/20/2014	0.3088
No. 2	(Third dose) 1 ds	3	12,817.2		4/202021	5/20/2014	0.7136 (Borderline)
No. 3	(Second dose) 313 ds	2	4869.6		6/1/2021	6/7/2017	0.8688 (Borderline)
No. 4	(Second dose) 153 ds	2	1812.7		12/30/2021	2/20/2015	0.1616
No. 5	(Second dose) 153 ds	2	139.3		12/30/2021	2/20/2015	0.4400

Note: Number of days (ds); Immunoglobulin (IgG).

## Data Availability

The data may be provided on reasonable request to the corresponding author.
